# Disability and Migration Routes: An Explorative Analysis Among Refugees Hosted in Italy

**DOI:** 10.3389/ijph.2024.1607821

**Published:** 2025-01-17

**Authors:** Marco Tofani, Maurizio Marceca, Donatella Valente, Giovanni Galeoto, Mohamed Ali Ben Zina, Imène Soumaya Salhi, Khadija Elmadmad, Hind Tak Tak, Justine Gosling, Satish Mishra, Valentina Gazzaniga, Marco Cilione, Silvia Iorio

**Affiliations:** ^1^ Department of Life Sciences, Health and Healthcare Professions, Università degli Studi “Link Campus University”, Rome, Italy; ^2^ Italian Society of Migration Medicine, Rome, Italy; ^3^ Department of Public Health and Infectious Diseases, Sapienza University of Rome, Rome, Italy; ^4^ Department of Human Neurosciences, Sapienza University of Rome, Rome, Italy; ^5^ Neuromed IRCCS, Pozzilli, Italy; ^6^ Faculty of Humanity at Tunis, University of Tunis, Tunis, Tunisia; ^7^ Faculty of Law Economics and Social Science, University Hassan II, Casablanca, Morocco; ^8^ UNESCO Center for Rights and Migration, Rabat, Morocco; ^9^ World Health Organization, Regional Office for Europe, Copenhagen, Denmark; ^10^ Department of Medico-Surgical Sciences and Biotechnologies, Sapienza University of Rome, Rome, Italy

**Keywords:** migrant and refugee health, disability, rehabilitation, mental health, functional limitations

## Abstract

**Objective:**

Data on disability in refugees is lacking, hindering effectiveness of humanitarian response. We investigated disability condition in refugees, identifying possible mechanisms that affect their health.

**Methods:**

The Washington Group Short Set – Enhanced was used to identify people at risk experiencing disability. Data on migration routes were collected and the relationship with functioning limitations was explored.

**Results:**

483 refugees (58.18% males - 41.82% female) were interviewed. 23.8% were found to have a disability, with a higher risk for those who travelled along the central Mediterranean route OR (95% CI) 2.08 (1.33–3.24). Affect domain represented the main weight for disability (28.16%), followed by mobility limitation (8.28%). People who travelled across the central Mediterranean route were found to have a high risk of developing anxiety problems OR (95% CI) 2.19 (1.33–3.6), while people who crossed the Balkan route had a higher risk of mobility limitation OR (95% CI) 3.03 (1.23–7.44).

**Conclusion:**

This study provides the first available data on disability among refugees in Italy, revealing a high prevalence of disability and a significant association with migration routes. These findings underscore the urgent need for targeted health and rehabilitation interventions to address the specific vulnerabilities of this population.

## Introduction

The World Report on Disability indicates that approximately 15% of the global population experiences some form of disability [1]. The United Nations (UN) Convention on the Rights of Persons with Disabilities (UNCRPD) [2] identifies people with disabilities as those with long-term physical, mental, intellectual, or sensory impairments that can impede their full, effective, and equitable participation in society.

Research on the prevalence of disability among refugees and asylum seekers is limited, with estimates ranging from 3%–10% [3, 4]. The term “migrant” lacks a universally accepted definition [5], but the United Nations Department of Economic and Social Affairs describes an international migrant as “any person who changes his or her country of usual residence” [6] due to conflict, economic issues, or environmental factors, encompassing individuals crossing international borders irrespective of their legal status. Specifically, asylum seekers and refugees are those seeking or granted international protection from another country.

Collecting data on refugees and asylum seekers is challenging due to the absence of documentation, such as medical histories and prior treatments [7], and barriers to accessing health and social care. Consequently, services often fail to meet the needs of these populations, adversely affecting their physical and mental health [8]. Universal health coverage remains a goal in many regions, with charitable organizations frequently attempting to bridge the gap. In the refugee and migrant population, physical health issues often arise from injuries, infectious diseases, and poorly managed chronic noncommunicable diseases [9, 10]. It is suggested that 1 in 6 refugees has a significant physical health problem impacting their quality of life [11], with musculoskeletal conditions and nonspecific pain among the most common, potentially benefiting from rehabilitation. Health screenings of refugees shortly after arrival reveal a high prevalence of noncommunicable diseases, which are challenging to manage and have long-term health implications warranting rehabilitation [10, 12].

Rehabilitation, defined as “a set of interventions designed to optimize functioning and reduce disability in individuals with health conditions in interaction with their environment” [13], is a crucial component of universal health coverage. It helps individuals maintain independence in daily activities and participate in education, work, recreation, and meaningful life roles [14].

In addition to physical health issues, refugees and asylum seekers are particularly vulnerable to mental health problems due to trauma experienced before, during, and after migration [15]. Estimates suggest that nearly two-thirds of refugees experience mental health issues such as anxiety, depression, and post-traumatic stress disorder [11, 16]. Social isolation, poverty, hostility, discrimination, and racism further exacerbate these mental health issues [17, 18]. Commonly reported problems include insomnia, memory disturbances, and difficulty concentrating, hindering adaptation in host communities [15]. Financial instability, safety concerns, future uncertainties, unemployment, and barriers to accessing services can worsen health problems, particularly mental health. There are also concerns about sexual, domestic, and gender-based violence, especially as refugees may be separated from family and have limited protection and community support [19].

Other health issues among refugees and migrants include nutritional deficiencies, infectious diseases, inadequate vaccination coverage, poor oral and eye health, and delays in child development milestones [19, 20]. Many migrants are unaware of available healthcare services, including rehabilitation and assistive technologies [21, 22]. Cultural differences and language barriers also hinder access to care, despite significant experience in addressing these challenges [23]. Barriers to healthcare access include a lack of funds, insufficient trained personnel, organizational inefficiencies, and poor coordination among service providers [24].

Cultural norms, healthcare-seeking behaviors, and the quality of health services differ between the country of origin and Italy, where universal health coverage exists, and international conventions protecting the rights of people with disabilities, migrants, and children have been ratified. To ensure the highest attainable health and protection levels for migrants with disabilities, comprehensive policies and frameworks on migrant rights and health must be developed at all system levels, including organizations representing persons with disabilities [25].

Experts advocate for improved service delivery models to address the health needs of refugees and asylum seekers [26], bridging gaps between identified needs and available services, including rehabilitation [27–30]. Comprehensive assessments of individual needs should be conducted by qualified personnel. Currently, no published models exist for meeting the rehabilitation needs of refugees, with varying needs and barriers across population groups and contexts, necessitating different services. This study aims to: a) estimate the proportion of refugees with disabilities; b) evaluate specific functional limitations in this population; and c) explore the relationship between migration routes and disability. The research aims to provide evidence to inform Italian policymakers about the rehabilitation needs of refugees, supporting the development of accessible, barrier-free services.

## Methods

### Tools

To assess disability and functionality among refugees and migrants, the research team utilized the Washington Group Short Set Enhanced (WG-SS-Enhanced). Developed and validated by the Washington Group on Disability Statistics (WG), this tool has been previously tested within refugee and asylum seeker populations in Italy [31]. The questions in the WG-SS-Enhanced reflect contemporary understandings of disability, employing the World Health Organization’s International Classification of Functioning, Disability, and Health (ICF) as its theoretical foundation [32]. This tool can be integrated into surveys focused on other topics, provided the survey design includes: a) detailed data collection on selected adult family members, and b) direct responses from participants, unless health issues prevent them from doing so. To ensure international comparability, the WG-SS-Enhanced collects information on difficulties individuals may have in performing basic functions, such as seeing, hearing, walking or climbing stairs, remembering or concentrating, self-care, communication (both expressive and receptive), upper body activities, and affective conditions like depression and anxiety. The WG-SS-E consists of 12 questions covering these eight functional domains. For this study, the Italian translation was used alongside English, French, Arabic, and Spanish versions as needed. The WG-SS-E was administered through interviews conducted either in person or via web calls, depending on participants’ availability and the constraints imposed by COVID-19 restrictions. Each interview lasted approximately 30–45 min. The tool was integrated into broader surveys designed to collect sociodemographic information and migration experiences, enabling a comprehensive understanding of disability within the participants’ broader contexts. Data collection took place from December 2020 to September 2022, allowing sufficient time to recruit a representative sample from multiple reception centers. This timeline was adjusted as needed to accommodate the operational capacities of the centers and the availability of participants. To ensure consistency and reliability, mediators followed a standardized protocol throughout the process. Data was captured using mobile devices and securely uploaded to a cloud server for analysis.

### Sampling and Procedures

To recruit participants, an initial email detailing the project’s objectives was sent to various stakeholders managing reception centers across the national territory. Since the Reception and Integration Service (SAI) centers are appointed by an Italian government agency known as the Central Service, official permission was requested to conduct interviews. Upon receiving this permission, the research team, along with social workers and cultural mediators, conducted the interviews. Depending on the availability of reception centers and COVID-19 restrictions, interviews were conducted either in person or via web calls.

Eligibility criteria for the study included both male and female participants, aged 18 or older, regardless of health status. Recruitment was structured to ensure representativeness without over-representation of specific subgroups, such as individuals with disabilities. To this end, the invitation to participate in the study did not specifically target people with disabilities. Instead, recruitment efforts were directed at all refugees hosted in participating reception centers, as determined by their availability and consent to participate. The interviews were scheduled based on the operational capacity of the reception centers and participants’ willingness to engage. Social workers and cultural mediators played a critical role in ensuring a fair and inclusive approach to participant selection by actively inviting all eligible individuals within the centers, irrespective of their health status or disability. Furthermore, the communication materials and consent processes explicitly emphasized that the study sought general insights from the refugee population and not specific data on disabilities. To calculate the sample size, it was considered that Italy hosts approximately 165,000 refugees [33], with an estimated 15% of this population living with disabilities. This calculation accounted for the general refugee population, not just those with disabilities, ensuring that the findings would reflect broader trends and experiences.

### Data Analysis

Data analysis was performed using the Statistical Package for the Social Sciences (SPSS) version 20.0 (Chicago, IL, United States). Sociodemographic characteristics were examined using frequency tables, mean values, and standard deviations (SD). Disability measurement adhered to the WG-SS-Enhanced standard threshold, where “a lot of difficulty” or “cannot do at all” in any domain, and for upper body functioning, “daily” and “a lot” in anxiety and depression, were considered [31]. The recommended SPSS syntax from the WG website for the WG-SS-Enhanced was utilized [34]. Contingency tables and Odds Ratios (ORs) were calculated for specific variables, such as gender and migration routes, with statistical significance set at p < 0.05 and 95% confidence intervals. This descriptive analysis focused on unadjusted associations to identify patterns in disability prevalence across migration routes. ORs were interpreted as measures of association, and no regression model was applied in the analysis. Data was collected using android tablets via a mobile application and transferred daily to a secure cloud server.

### Risks of Bias

The WG-SS Enhanced is available in multiple languages. To mitigate comprehension issues, even for those not fluent in these languages, language mediators were involved. These mediators, after receiving 1-day training (either in-person or online) and reviewing the tool, assisted during interviews.

### Ethical Considerations

Ethical approval was granted by the Department of Human Neurosciences, Sapienza University of Rome (Code N1311 30 June 2021). Written informed consent was obtained from all participants aged 18 and above. Participants identified with specific health needs, including rehabilitation and mental health services, were referred to local health authorities. Information about locally available services was also provided to participants experiencing disabilities.

## Results

The study sample included 483 individuals with an average age of 30.12 years (SD 8.45). Of these, 21.5% had no formal education, 21.1% had attended some level of college or school, and 19% had vocational training. A majority of 55.9% reached Italy via the central Mediterranean route, while 20% used the western Mediterranean route. Detailed sociodemographic characteristics and additional information about the participants can be found in Table 1.

**TABLE 1:** Sociodemographic characteristics of the sample (total 483) (Italy, 2023).

Age mean (SD)	30.12 (8.45)	
Sex	N	%
Female	202	41.82
Male	281	58.18
Education	N	%
No schooling or never completed any grade	104	21.53
Primary school	65	13.46
Vocational Education	92	19.05
Professional training	22	4.55
Secondary school	91	18.84
College	102	21.12
University	7	1.45
Migration Routes	N	%
Western Mediterranean route	97	20.08
Central Mediterranean route	268	55.49
Eastern Mediterranean route	16	3.31
Balkan route	36	7.45
Humanitarian corridors	5	1.04
From Ukraine^a^	60	12.63
Distribution of Reception Centers in Italy	N	%
North	54	11.17
Center	253	53.38
South and Islands	176	36.44

^a^ Please note that after the invasion of Ukraine, it was necessary include a specific data entry to include these people, despite it is not a migration routes.

The prevalence of disability within the sample, as defined by significant difficulty or inability in any WG-SS Enhanced domain and/or symptomatic anxiety and depression, was calculated at 23.81% (95% CI 20.01–27.61). The most common functional impairments were anxiety and depression, affecting 18.22% (95% CI 14.78–21.66) and 9.94% (95% CI 7.27–12.60) of the sample, respectively. Table 2 provides a breakdown of disability prevalence and specific functional limitations. Additionally, the analysis revealed that women were more likely than men to experience disability (OR 1.38–95% CI 0.9–2.1).

**TABLE 2:** Proportion of people with disability and functional limitations (Italy, 2023).

	N	%	95% CI
Vision	17	3.52	2.06-5-58
Hearing	6	1.24	0.04–2.68
Mobility	40	8.28	5.98–11.1
Communication	24	4.97	3.03–7.30
Self-care	12	2.48	1.19–4.30
Cognition	23	4.76	2.86–6.66
Upper Body	20	4.14	2.36–5.92
Anxiety	88	18.22	14.78–21.66
Depression	48	9.94	7.27-12-6
People with disability	115	23.81	20.01–27.61

	With Disability n(%)	Without Disability n (%)	OR 95% CI
Men	60 (52.18)	221 (60.06)	1.00
Women	55 (47.82)	147 (39.94)	1.38 (0.9–2.1)

Given that anxiety, depression, and mobility issues were the primary contributors to the observed disability rates, the study further explored their association with different migration routes. The results are detailed in Table 3.

**TABLE 3:** Individual level characteristics and risk across migration routes (Italy, 2023).

	With disability	%	Without disability	%	OR (95% CI)
Other routes	36	31.31	179	48.65	1.00
CM Route	79	68.69	189	51.35	2.08 (1.33–3.24)

	Anxiety Yes	%	Anxiety No	%	OR (95% CI)
Other routes	26	29.55	189	47.85	1.00
CM Route	62	70.45	206	52.15	2.19 (1.33–3.6)

	Depression Yes	%	Depression No	%	OR (95% CI)
Other Routes	22	45.84	193	44.37	1.00
CM Route	26	54.16	242	55.63	0.94 (0.52–1.71)

	Mobility Limitation Yes	%	Mobility Limitation No	%	OR (95%CI)
Other Routes	33	82.50	414	93.46	1.00
Balkan Route	7	17.50	29	6.54	3.03 (1.23–7.44)

CM route, Central Mediterranean route.

## Discussion

According to the study sample, 23.8% (20.05–27.61 95% CI) of refugees experienced disabilities, a figure higher than the global average of 15%. Women in the sample were found to be 38% (OR 1.38) more likely to experience disabilities compared to men. This aligns with prior research [31] indicating a higher incidence of disability among women, possibly due to their increased risk of physical and sexual violence [35, 36]. In terms of specific disability domains, anxiety disorders were most prevalent (18.22%), followed by depression (9.94%) and mobility limitations (8.28%). These results contrast with the WHO rehabilitation needs report in Italy [37], which identified musculoskeletal disorders as the most common condition. Similarly, Doocy and colleagues [38] reported a higher prevalence of musculoskeletal conditions, leading to a greater incidence of mobility-related disabilities. The discrepancy may be attributed to differing methodologies; for instance, Doocy’s study utilized a biomedical model focusing on bodily functions and structures as defined by the ICF, possibly underestimating mental health-related disabilities. In contrast, the WG-SS-E approach includes an assessment of activities and mental health functions, which are crucial for understanding migrants’ health [31, 39]. It is well-established that populations affected by armed conflict are exposed to traumatic events and chronic stressors, resulting in elevated mental health issues [40, 41]. Data from studies on internally displaced adults in Georgia [42] and Syrian refugees in Turkey [43] indicate high levels of anxiety and depression, reflecting the significant mental health burden in conflict-affected displaced populations. The WHO draft decision, supported by several Member States [44], underscores the importance of integrating mental health support within rehabilitation services to achieve universal health coverage.

A novel aspect of our study was the exploration of disability prevalence based on migration routes. Initially, we categorized refugees and asylum seekers by migration routes, but the Russian invasion of Ukraine on February 24, 2022, necessitated the inclusion of this population. Nearly 172,000 Ukrainians fleeing the conflict have arrived in Italy [45], prompting us to create a specific category for them. Our findings indicate significant variations in disability prevalence based on migration routes. Individuals arriving via the Central Mediterranean route faced twice the risk of disability (OR 2.2) compared to those using other routes. This route, particularly through Libya, is notorious for its dangers, including documented instances of violence [46]. Women, especially, are at heightened risk of sexual violence, and healthcare access in Libya is severely limited [47], highlighting the urgent need for psychosocial support. Consistent with our previous findings (OR 1.58) [31], women in this sample experienced disabilities more frequently than men (OR 1.38), likely due to higher violence exposure [36, 47].

Examining the impact of migration routes on mental health, those who traveled through the Central Mediterranean route had a significantly higher risk of anxiety disorders (OR 2.19), though no significant difference was found in depression rates (OR 0.94) between routes. High levels of violence exposure among migrants correlate with increased mental health disorders, including PTSD and major depressive disorder [48], as supported by multiple studies [43, 48]. Regarding mobility limitations, individuals traveling the Balkan route were three times more likely to experience functional mobility issues (OR 3.03, 95% CI 5.98–11.1). Research by ben Farhat and colleagues found that nearly one-third of migrants/refugees on this route experienced violent events, including physical trauma [49].

It is important to point out that the WG-SS-E focuses on identifying activity limitations rather than determining the timing or cause of specific health conditions. As such, this tool assesses current functional difficulties impacting daily life, regardless of whether they originated before, during, or after migration. While the timing of causes of health condition may offer additional insights, the findings aim to highlight the prevalence of functional limitations among refugees to inform targeted health and rehabilitation interventions. Given this evidence, in fact, it is crucial for stakeholders to design targeted services for these vulnerable populations and address barriers to accessing health and rehabilitation services, including assistive technologies. The availability of rehabilitative services like occupational therapy, physiotherapy, and speech and language therapy for refugees with disabilities is limited, often not included in primary healthcare services [50–52]. Barriers such as unstructured healthcare services, financial constraints, fluctuating healthcare needs, a shortage of experienced healthcare professionals, and cultural and language differences further restrict healthcare access for refugees [53]. Additionally, challenges in social integration, livelihood, and empowerment are significant [54], necessitating multi-sectoral and cross-cutting strategies from governments and stakeholders.

### Limitations

The study found a high proportion of refugees experiencing disabilities, with significant variation based on migration routes. However, several limitations and potential biases should be considered. Sampling bias may exist due to the reliance on reception-center-based recruitment, and self-reporting bias could influence the accuracy of disability prevalence estimates. The COVID-19 pandemic imposed restrictions on face-to-face interviews, necessitating the use of web-based data collection methods in some cases. This shift introduced challenges related to internet connectivity and digital literacy, which may have influenced the quality of the data collected. Furthermore, language barriers posed difficulties despite the availability of translated tools and trained mediators. These factors, along with operational challenges within reception centers and participants’ psychosocial stress, may have affected response consistency and reliability. Efforts to mitigate these biases, including mediator training and standardized protocols, were implemented but cannot entirely eliminate these risks.

This study calculated unadjusted ORs to explore the relationship between migration routes and disability conditions. While ORs are commonly used for such analyses, they may overestimate relative risks. The exploratory design of the study prioritized identifying patterns over causal inference, and no multivariable adjustments were made. Future research could incorporate regression models or alternative approaches, such as log-binomial models, to refine risk estimates and account for potential confounding factors. Furthermore, the study did not examine disability among unaccompanied minors and children, who constitute a significant portion of refugees. Future research should focus on assessing disabilities in children using specific tools like the UNICEF and WG-developed Child Functioning Module [55]. Additionally, the study only included individuals with refugee or asylum seeker status, excluding many others who lack legal status but likely live with disabilities. Future studies should find ways to include these individuals and evaluate their needs. Further research should also investigate the barriers refugees face in navigating the national health system and the capabilities of local health authorities in reaching this population.

### Conclusions

The study found a high proportion of refugees experiencing disabilities, with significant variation based on migration routes. Local and national stakeholders must develop strategies to address the rehabilitation and assistive technology needs of refugees with disabilities and reduce barriers to accessing these essential services.
